# Evaluation of the Effect of Various Beverages and Food Materials on the Color Stability of Provisional Materials: An In Vitro Study

**DOI:** 10.7759/cureus.63941

**Published:** 2024-07-06

**Authors:** Tanveer Fatima, Parvez Abubakar, Sumit Deshpande, Nazia Afreen, G Sheshnag, Syeda Safoora

**Affiliations:** 1 Department of Prosthodontics, Al-Badar Rural Dental College and Hospital, Kalaburagi, IND

**Keywords:** color stability, beverages, bis-acryl composite, polymethyl methacrylate, ivoclar vivadent, provisional restorative materials

## Abstract

Aim

This study aims to evaluate the color stability of four provisional materials: polymethyl methacrylate (DPI® Self-Cure), 10-ethoxylated bisphenol A dimethacrylate (Oratemp® C&B), bis-acryl composite resin (Systemp® C&B, Ivoclar Vivadent), and bis-acryl composite (Systemp® C&B, Ivoclar Vivadent) combined with light-cure composite (Fusion Flo® LC).

Materials and methods

A total of 40 specimens were meticulously crafted from modeling wax into discs, each precisely 2 mm thick and 20 mm in diameter. Four provisional materials were packed into molds, yielding 10 specimens for each material group. After mixing and polymerization, the specimens were trimmed and polished. Reflectance spectrophotometers were used for initial color assessments based on the CIELAB color space system. Staining solutions, including coffee, Tata Green Tea, Pepsi, and turmeric, were prepared to mimic dietary agents. Artificial saliva, replicating oral conditions, was formulated and sterilized. The specimens were then immersed in various solutions for 15 days at 37 °C. Color measurements were taken on days 2 and 15 using the same spectrophotometer, calculating color differences (ΔE) from changes in L*, a*, and b* values.

Results

DPI Self-Cure (polymethyl methacrylate) was found to be the most color-stable temporary restorative material, followed by Vivadent (bis-acryl composite resin), Oratemp (10-ethoxylated bisphenol A dimethacrylate), and Fusion Flo (light-cure composite). Fusion Flo exhibited the highest color change by the 15th day. Coffee and green tea demonstrated the greatest potential for causing color changes in the provisional restorative materials.

Conclusion

DPI Self-Cure exhibited the highest color stability among the provisional materials, with Vivadent and Oratemp following closely behind. Green tea and coffee were the most potent staining agents, while Pepsi and turmeric induced lesser color changes.

## Introduction

Provisionalization, also known as temporization, constitutes a critical phase within prosthodontics, serving to temporarily enhance aesthetics, stabilize function, or both for a finite period until the definitive or permanent prostheses can be installed. This intermediary step holds particular significance in cases where patients require dental restorations that necessitate time-consuming processes, such as the fabrication of crowns, bridges, or implants. During this provisional phase, patients expect not only functional stability but also an aesthetically pleasing appearance that closely resembles their natural dentition [1].

In the context of aesthetic dentistry, provisional restorations must fulfill several essential criteria to meet patient expectations. Firstly, they must provide an initial shade match that harmonizes with the surrounding dentition, ensuring a seamless transition between the provisional and final restorations. Additionally, maintaining this aesthetic appearance over the duration of their service is paramount. Any discernible changes, particularly in color, can undermine the acceptability of provisional restorations and lead to patient dissatisfaction.

One of the primary concerns regarding provisional restorations is the potential for color change or discoloration over time. This phenomenon can arise due to various factors, such as chemical interactions with oral fluids, exposure to environmental elements, or the ingestion of pigmented food and beverages [2]. As provisional restorations are often in place for several weeks or months, ensuring their color stability is crucial to maintaining patient satisfaction and preventing the need for premature replacement, which can entail additional costs and inconvenience [3].

Color stability has emerged as a significant consideration in the selection of provisional materials, particularly in cases where aesthetics play a central role [4]. While a plethora of studies in the literature have endeavored to assess the color stability of different temporary restorative materials, the findings have sometimes been conflicting or inconclusive. This discrepancy underscores the need for comprehensive and comparative research to provide clinicians with reliable guidance in material selection.

The present study seeks to address this gap by conducting an analysis of the color stability of four commonly utilized provisional restorative materials. By subjecting the materials to immersion in various beverages and food substances, the study aims to evaluate the color stability of four provisional materials: polymethyl methacrylate (DPI® Self-Cure), 10-ethoxylated bisphenol A dimethacrylate (Oratemp® C&B), bis-acryl composite resin (Systemp® C&B, Ivoclar Vivadent), and bis-acryl composite (Systemp® C&B, Ivoclar Vivadent) combined with light-cure composite (Fusion Flo® LC).

## Materials and methods

Methodology

A total of 40 specimens were prepared for this study using modeling wax carved into disc shapes, each precisely 2 mm thick and 20 mm in diameter. The preparation involved three main steps: shaping the wax into discs, flasking to support the wax molds, and dewaxing to create the final molds. These molds were then packed with four different provisional materials selected for this study. Following manufacturer guidelines, the provisional materials were mixed and polymerized, then trimmed using tungsten carbide acrylic burs and polished with pumice powder for a smooth finish. Color assessments were conducted using a reflectance spectrophotometer in the CIELAB color space system to measure parameters L*, a*, and b*. Staining solutions were prepared to simulate dietary agents: coffee, Pepsi, green tea, and turmeric, along with artificial saliva. The specimens were immersed in these solutions and artificial saliva for 15 days at 37 °C, with color measurements taken initially (T2) and after 15 days (T15) to assess color stability. The solutions were not changed during the entire duration of the study. This allows us to observe the cumulative effects of the solutions on the color stability of the dental materials over time. The ΔE values were calculated to determine color changes (ΔL*, Δa*, Δb*) between the two time points, ensuring a consistent methodology across all specimen groups.

Specimen preparation

**Figure 1:**
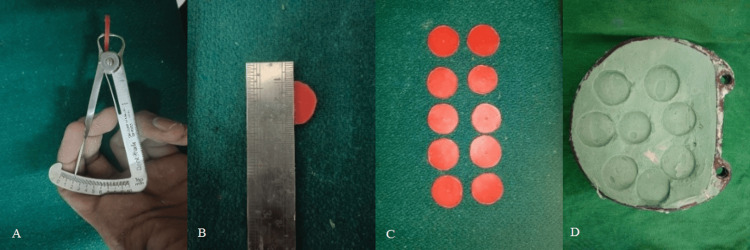
Specimen preparation. (A) 2 mm thickness using wax gauge into disc; (B) 20 mm diameter disc; (C) modeling wax carved; (D) mold obtained after wax elimination

**Figure 2:**
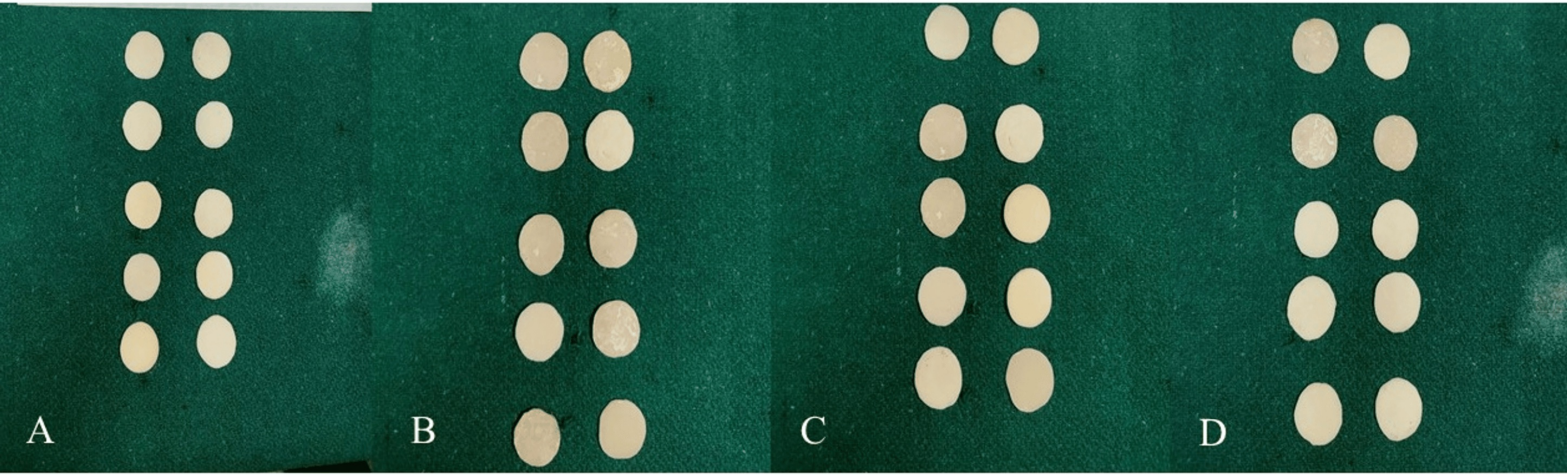
Specimens of four different provisional materials. (A) polymethyl methacrylate (DPI Self-Cure); (B) 10-ethoxylated bisphenol A dimethacrylate (Oratemp C&B); (C) bis-acryl composite resin (Vivadent); (D) light-cure composite (Fusion Flo)

A total of 40 specimens were prepared using modeling wax carved into discs measuring precisely 2 mm thick and 20 mm in diameter. The process began with three primary steps: Firstly, modeling wax was meticulously shaped into the desired disc forms. Subsequently, these wax discs underwent flasking, wherein they were enclosed in a flask to provide structural support for ensuing procedures. Following this, the flasked discs underwent dewaxing, a crucial step involving wax removal to yield the molds required for specimen creation. Finally, the molds were revealed upon opening the flask and primed for packing with provisional materials (Figures 1, 2).

Provisional material preparation

Four different provisional materials were selected for this study, each packed into the molds to create the sample specimens. Each material group consisted of 10 specimens, making up the following groups: Group 1: polymethyl methacrylate (DPI), Group 2: 10-ethoxylated bisphenol A dimethacrylate (Oratemp C&B), Group 3: bis-acryl composite resin (Systemp C&B, Ivoclar Vivadent) and Group 4: bis-acryl composite (Systemp C&B, Ivoclar Vivadent) combined with light-cure composite (Fusion Flo LC).

Specimen processing

Following the manufacturer's instructions, all provisional materials underwent meticulous mixing to guarantee precise chemical consistency and optimal performance. Once the polymerization was complete, the specimens were carefully trimmed using green-coded tungsten carbide acrylic trimming burs. Subsequently, polishing was carried out using pumice powder in a wet buff to attain a smooth and flawless surface finish. The uniformity of the specimens during trimming and polishing was maintained using a gauge. Gross trimming ensured that the specimens were cut to similar sizes, while the gauge was used to achieve consistent thickness and surface finish across all specimens.

Baseline color measurement

The initial color assessments of all specimens were conducted utilizing a reflectance spectrophotometer (FRU, Shenzhen, China), operating within the framework of the CIELAB color space system. This system delineates color through three fundamental parameters: L (representing lightness), a (reflecting the spectrum from green to red), and b (encompassing the spectrum from blue to yellow).

Artificial saliva preparation

To replicate the conditions of the oral environment, a laboratory-produced artificial saliva was formulated. The pH of the solution was carefully adjusted to 6.75 ± 0.15 by adding 10 N sodium hydroxide. Subsequently, the final mixture underwent sterilization in an autoclave to eliminate any potential contaminants, ensuring its suitability for use in the study [3].

Preparation of the staining solutions

To emulate typical dietary staining agents, the staining solutions were prepared at the following concentrations: Tata Green Tea was prepared by incorporating 2.8 g of tea leaves into 150 mL of boiling distilled water. Coffee (Nescafe, Nestle, Gurugram, India) involved adding 2.8 g of coffee to 150 mL of boiling distilled water. The turmeric solution was prepared by adding 0.5 g of turmeric to 150 mL of boiling distilled water. Pepsi was employed in its original state as supplied by Pepsico India Holdings Pvt. Ltd. (Nelamangala, India).

First, 150 mL of each staining solution was prepared to ensure proper mixing. This mixture was then diluted with additional distilled water to reach the required volume (330 mL). The solutions were then mixed with 660 mL of artificial saliva to obtain a standard 1-L solution. The ratio of beverages to artificial saliva was determined based on the average daily saliva secretion, which ranges from 0.5 to 1.5 L. Therefore, to simulate this natural condition, the study used 660 mL of artificial saliva as a baseline. To complete the 1-L mixture, 330 mL of the beverages were added. This ratio was selected to closely mimic the dilution and interaction of beverages with saliva in the oral environment, providing a realistic assessment of the color stability of the dental materials under conditions similar to those in everyday use.

Immersion in staining solutions

To evaluate the color stability of the provisional materials in different staining solutions, the specimens were subdivided into five subgroups and immersed in the following mixtures: Subgroup I was a blend of coffee (330 mL) with artificial saliva (660 mL); Subgroup II was a fusion of Pepsi (330 mL) with artificial saliva (660 mL); Subgroup III was a combination of turmeric solution (330 mL) with artificial saliva (660 mL); Subgroup IV was a concoction of green tea (330 mL) with artificial saliva (660 mL); and Subgroup V consisted of only artificial saliva, serving as the control group.

Each specimen was immersed in its respective solution and kept at a constant temperature of 37 °C to simulate the oral environment. The specimens were immersed for a total of 15 days, aligning with the maximum duration for which a provisional restoration is typically used. Color measurements were taken on the 2nd and 15th day using the same reflectance spectrophotometer. The color difference (ΔE) for each specimen was calculated by measuring the changes in L*, a*, and b* values (ΔL, ΔA, and ΔB) at each time interval (T2 and T15).

## Results

Table 1 shows the color stability (ΔE) of Vivadent, Self-Cure, Oratemp, and Fusion Flo under two distinct conditions. Firstly, the samples were initially polished (day zero), and secondly, they were subjected to exposure to saliva for a duration of 15 days. Upon initial assessment (day zero), Vivadent and Self-Cure exhibited relatively minor color changes, with ΔE values of 1.87 and 1.85, respectively. Oratemp demonstrated even less color alteration, registering a ΔE of 0.32. In contrast, Fusion Flo showed a more pronounced color shift, with a ΔE value of 2.55. Following exposure to saliva for 15 days, Vivadent displayed the slightest color change, with a ΔE value of 0.25, suggesting excellent color stability. Self-Cure and Fusion Flo also maintained relatively stable colors, with ΔE values of 0.44 and 0.39, respectively. However, Oratemp experienced a significant increase in color difference, with a ΔE value of 5.30, indicating notable color alteration after exposure to saliva (Table 1).

**Table 1:** Influence of initial polishing and saliva exposure on the color stability of dental materials Vivadent: bis-acryl composite resin; Oratemp: 10-ethoxylated bisphenol A dimethacrylate; DPI Self-Cure: polymethyl methacrylate; Fusion Flo: bis-acryl composite combined with light-cure composite; L: lightness; a: red/green coordinate; b: yellow/blue coordinate; ΔE: color difference

Sl. No.	Sample Details	Test Parameter	Results			
			L	a	b	ΔE
1	Only polished day zero (standard group)					
	Vivadent	Customer specification, VMS/MECH/SOP/01	62.75	2.08	13.59	1.87
	DPI Self-Cure		76.33	-0.98	9.55	1.85
	Oratemp		58.07	-0.56	5.84	0.32
	Fusion Flo		61.07	-1.21	-2.92	2.55
2	Only in saliva for 15 days (standard group)					
	Vivadent	Customer specification, VMS/MECH/SOP/01	75.12	-0.80	9.61	0.25
	DPI self-Cure		75.87	-0.85	0.44	0.44
	Oratemp		74.65	0.01	5.30	5.30
	Fusion Flo		86.77	-0.05	0.39	0.39

The color stability of four different dental materials (Oratemp, Self-Cure, Vivadent, and Fusion Flo) after exposure to four distinct staining agents (coffee, Pepsi, turmeric, and green tea) over two time points (2nd day and 15th day) with color changes measured using ΔE values are presented in Table 2. Oratemp exhibits consistent color stability across most staining agents between the 2nd and 15th day, with relatively low ΔE values indicating minimal color changes. Notable exceptions include green tea, where a considerable increase in ΔE values suggests significant color alteration over the 15-day period. DPI Self-Cure demonstrates overall stable coloration, with minor variations in ΔE values observed across different staining agents between the two time points. Vivadent exhibits moderate to significant color changes in response to certain staining agents, as evidenced by a substantial decrease in ΔE values between the 2nd and 15th day. However, it is worth noting that the Pepsi group shows an increase in ΔE values over time, indicating a contrary trend compared to the other staining agents. Fusion Flo displays varying degrees of color stability across staining agents, with notable changes in ΔE values between the 2nd and 15th day. The most substantial color alterations occur in the coffee and Pepsi staining groups, reflecting a higher susceptibility to staining than the other materials (Table 2).

**Table 2:** Influence of various beverages on the color stability of dental materials on the 2nd and 15th days Vivadent: bis-acryl composite resin; Oratemp: 10-ethoxylated bisphenol A dimethacrylate; DPI Self-Cure: polymethyl methacrylate; Fusion Flo: bis-acryl composite combined with light-cure composite; L: lightness; a: red/green coordinate; b: yellow/blue coordinate; ΔE: color difference

Sl. No.	Sample Details	Results							
		Day 2				Day 15			
		L	a	b	ΔE	L	a	b	ΔE
1	Oratemp								
	Coffee	63.75	2.52	9.14	0.21	62.74	1.14	7.71	0.78
	Pepsi	61.34	0.62	6.53	1.13	63.51	0.92	6.26	1.40
	Turmeric	65.09	1.30	10.45	0.68	66.87	0.49	8.12	0.76
	Green tea	59.88	2.48	12.29	0.26	69.12	2.56	9.21	5.11
2	DPI Self-Cure								
	Coffee	77.02	-0.28	13.80	0.21	75.85	-0.17	10.81	0.78
	Pepsi	75.87	-0.58	9.67	0.45	76.30	-0.31	10.56	0.66
	Turmeric	77.89	0.25	14.30	0.23	80.31	0.81	12.88	0.24
	Green tea	75.88	-0.53	10.76	0.26	76.83	-0.08	11.52	0.70
3	Vivadent								
	Coffee	73.70	2.56	8.02	1.00	72.48	2.06	6.85	0.85
	Pepsi	65.56	0.29	7.28	1.20	73.40	0.56	7.53	1.70
	Turmeric	72.58	-0.21	7.91	0.72	72.10	0.71	9.44	0.32
	Green tea	62.29	7.91	12.78	3.25	71.36	3.26	14.94	1.44
4	Fusion Flo								
	Coffee	73.17	0.30	2.98	1.28	58.71	1.33	-0.35	11.21
	Pepsi	76.85	-0.88	9.64	0.30	55.53	-0.34	-1.31	5.43
	Turmeric	61.61	-0.01	6.88	2.50	64.68	-0.94	7.03	1.83
	Green tea	65.83	-0.18	3.47	1.45	60.86	4.35	13.18	3.24

In this study, the ΔE value, with a threshold of ΔE<3.7, serves as the clinically accepted stability criterion for restorative materials, where smaller ΔE values indicate greater acceptability of color changes in the material. Notably, DPI Self-Cure, consisting of polymethyl methacrylate, exhibited the slightest color change on the 15th day compared to any other material tested. Conversely, Oratemp demonstrated the highest color change, particularly when exposed to the green tea solution (ΔE difference = 4.85). Fusion Flo, composed of bisacryl composite (system C and B, Ivoclar Vivadent), exhibited the highest color change when exposed to coffee (ΔE difference = 9.93) and Pepsi (ΔE difference = 5.23) on the 15th day (Table 3).

**Table 3:** Differences in color stability of dental materials in various staining solutions Vivadent: bis-acryl composite resin; Oratemp: 10-ethoxylated bisphenol A dimethacrylate; DPI Self-Cure: polymethyl methacrylate; Fusion Flo: bis-acryl composite combined with light-cure composite; ΔE: color difference

Solution	Material ΔE											
	Oratemp			DPI Self-Cure			Vivadent			Fusion Flo		
	Day 2	Day 15	ΔE difference	Day 2	Day 15	ΔE difference	Day 2	Day 15	ΔE difference	Day 2	Day 15	ΔE difference
Standard group	Polished	Saliva	4.98	Polished	Saliva	-1.45	Polished	Saliva	-1.62	Polished	Saliva	-2.16
	0.32	5.30		1.85	0.44		1.87	0.25		2.55	0.39	
Coffee	0.21	0.78	0.57	0.21	0.78	0.57	1.00	0.85	-0.15	1.28	11.21	9.93
Pepsi	1.13	1.40	0.27	0.45	0.66	0.21	1.20	1.70	0.5	0.30	5.43	5.23
Turmeric	0.68	0.76	0.08	0.23	0.24	0.01	0.72	0.32	-0.4	2.50	1.83	-0.67
Green tea	0.26	5.11	4.85	0.26	0.70	0.44	3.25	1.44	-1.81	1.45	3.24	1.79

## Discussion

The evaluation of discoloration in dental materials represents a crucial aspect of assessing their suitability for clinical use. Traditionally, such assessment has relied on visual comparison, which, while widely employed, is susceptible to inconsistencies due to variations in color perception among different observers [5]. To overcome this limitation, instrumental techniques such as spectrophotometers and colorimeters have been increasingly utilized [6,7]. Among these instruments, spectrophotometers are considered superior due to their ability to measure the reflectance curve of a product's color at intervals as fine as 10 nm or less, thereby providing more accurate and precise color data [8]. Different studies have established varying thresholds for perceptible color differences, with ΔE values ranging from 1 to above 3.7 [5,9,10]. A ΔE value below 3.7 is generally considered clinically acceptable [11].

This study comprehensively evaluated the color stability of four provisional dental materials, Oratemp, Self-Cure, Vivadent, and Fusion Flo, when exposed to common staining solutions such as coffee, Pepsi, turmeric solution, and green tea over a period of 15 days. The ΔE values, which denote color differences, were meticulously measured on the 2nd and 15th days to determine the extent of discoloration each material experienced. These four materials were selected due to their ease of use and cost-effectiveness. The study evaluated the color stability of provisional dental materials on the 2nd and 15th days for specific reasons: the 2nd-day measurement provided an early assessment after exposure to staining agents, capturing immediate effects, while the 15th-day assessment represented the maximum recommended duration for these temporary materials in clinical use [12].

In the context of the present study, the color stability of provisional dental materials was investigated. DPI Self-Cure, primarily composed of polymethyl methacrylate, emerged as the material with the most robust color stability. Vivadent followed closely behind, exhibiting relatively stable color characteristics. In contrast, Fusion Flo displayed the least resistance to color change over time. This variation in color stability may be attributed to differences in the resin matrix and porosity among the materials. Polymethyl methacrylate likely possesses a more stable structure than Fusion Flo, contributing to its superior color stability.

DPI Self-Cure emerged as the most color-stable material overall. Specifically, it demonstrated remarkable resistance to discoloration caused by turmeric and green tea, showing only minor changes in color. This robustness makes DPI Self-Cure particularly suitable for clinical use, where prolonged exposure to staining agents is a concern. Vivadent, on the other hand, exhibited moderate color stability. While it maintained its color well in some solutions, its performance varied, indicating that it may be more suitable for shorter-term applications or environments with less exposure to strong staining agents.

Fusion Flo stood out for its poor color stability. The material underwent significant color changes, particularly when immersed in coffee and Pepsi. This suggests that Fusion Flo may be less ideal for provisional restorations in patients who consume these beverages regularly. Oratemp showed good overall color stability with most solutions, although it was notably affected by green tea, which caused substantial discoloration. This indicates that while Oratemp can be a reliable choice, it might not be the best option for patients who consume large amounts of green tea.

Among the staining agents tested, green tea and coffee were the most potent, causing the highest ΔE values across several materials. Green tea, in particular, emerged as the strongest staining agent, leading to significant color changes in all tested materials. Coffee also caused considerable discoloration but to a slightly lesser extent. Pepsi and turmeric solutions, while still causing some degree of color change, were less aggressive in their staining effects than green tea and coffee.

The findings of the present study correlate with those of Mutlu-Sagesen et al., who evaluated the color change of reinforced acrylic, porcelain, and conventional acrylic teeth after immersion in tea, coffee, and cola and found similar results [13]. Similarly, Koksal and Dikbas observed significant color changes in acrylic teeth and porcelain after immersion in cola, coffee, and tea for one week, two weeks, and one month [14]. This is in contrast to Mousavi et al.'s [15] study, which investigated the color stability of three acrylic tooth brands exposed to coffee, tea, and cola. Coffee caused significant color changes in apple acrylic teeth, while tea induced uniform changes across all groups. Cola resulted in the greatest color alteration in Ivoclar teeth.

The discoloration of resins by fluids and beverages is primarily caused by the adsorption or absorption of colorants present in these substances [16]. Our study identified green tea as the most powerful staining agent, resulting in the most significant color changes in the tested materials. The strong staining ability of green tea can be attributed to its chromogenic compounds, such as tannins and catechins, which have a high affinity for resin-based materials. The staining process varies depending on the source of the colorant, with tea and coffee exhibiting different mechanisms of discoloration. According to Um and Ruyter [7] and Hersek et al. [17], tea staining is primarily attributed to the adsorption of polar colorants, while coffee staining results from both surface adsorption and absorption of colorants. This differential mechanism of discoloration may explain why coffee exhibits lower color stability than tea in certain dental materials. The staining effect of coffee is likely due to its tannins and polyphenolic compounds, which can infiltrate the porous surfaces of dental materials, leading to discoloration. Pepsi, which contains caramel color and acidic components, also caused color changes in the materials, but its staining impact was not as significant as that of green tea and coffee.

Based on the findings of the study, DPI Self-Cure polymethyl methacrylate emerges as the preferred choice for provisional restorations lasting up to 15 days in clinical practice. Its superior color stability makes it well-suited for prolonged use in temporary restorations. Conversely, Vivadent may be more suitable for shorter-term applications, such as those lasting seven days or less. These recommendations underscore the importance of considering material properties and application duration when selecting provisional restorative materials in clinical settings. Additionally, surface characteristics of dental materials, including wear resistance, roughness, and polishability, also play a significant role in determining color stability. Materials with smoother surfaces are less prone to staining and discoloration. Future studies could investigate the impact of these surface properties on color stability to enhance our understanding of the factors influencing discoloration in dental materials.

The study's strength is that it rigorously evaluates specific provisional dental materials under controlled conditions, focusing on practical outcomes such as color stability over crucial time intervals (2nd and 15th days). Its systematic approach enhances the understanding of material performance in clinical settings, offering actionable insights for dental professionals in material selection and patient care. Moreover, it prepares for real-world scenarios where patients might not adhere to timelines, ensuring clinicians are equipped to manage prolonged exposure to these materials in practice.

The limitation of the study is that the study was conducted in a controlled laboratory setting, which may not fully replicate the conditions encountered in clinical practice. Factors such as temperature fluctuations, pH variations, and oral hygiene practices could influence color stability differently in vivo. Additionally, the study evaluated color stability over a relatively short duration, focusing only on the 2nd and 15th days. Long-term color stability beyond this timeframe remains uncertain and warrants further investigation, especially since patients might not adhere to recommended timelines. Furthermore, the study utilized specific brands and formulations of provisional materials and staining solutions, which may not fully represent the wide array of products available in clinical settings. Variations in material composition and staining agents could yield different results. Lastly, the study primarily relied on instrumental color measurement techniques, while visual assessment by clinicians and patients may offer additional perspectives on color stability and acceptability.

## Conclusions

DPI Self-Cure, containing polymethyl methacrylate, displayed the highest color stability among provisional materials, showcasing its robust resistance to color changes. Following DPI Self-Cure, Vivadent and Oratemp demonstrated commendable color stability, albeit slightly lower. Notably, our study highlighted green tea as the most potent staining agent, causing the most significant color alterations in the tested materials. Coffee closely followed green tea in its staining propensity. Additionally, Pepsi and turmeric solutions also induced color changes, although to a lesser extent than green tea and coffee. These findings provide valuable insights into the color stability of provisional materials and the staining potential of common beverages, aiding clinical decision-making processes.
